# External validation and comparative performance of the SLANT score for neuroprognostication in out-of-hospital cardiac arrest survivors undergoing targeted temperature management: insights from an Asian cohort

**DOI:** 10.1186/s40560-025-00778-y

**Published:** 2025-02-14

**Authors:** Yi-Ju Ho, Cheng-Yi Fan, Yi-Chien Kuo, Chi-Hsin Chen, Chun-Ju Lien, Chun-Hsiang Huang, Chien-Tai Huang, Sih-Shiang Huang, Ching-Yu Chen, Chih-Wei Sung, Wen‑Chu Chiang, Wei-Tien Chang, Chien-Hua Huang, Edward Pei-Chuan Huang

**Affiliations:** 1 Department of Emergency Medicine, National Taiwan University Hospital, Taipei, Taiwan; 2 Department of Emergency Medicine, National Taiwan University Hospital Hsin-Chu Branch, Hsinchu, Taiwan; 3 Department of Emergency Medicine, National Taiwan University Hospital Yun-Lin Branch, Yunlin, Taiwan; 4 Department of Emergency Medicine, College of Medicine, National Taiwan University, Taipei, Taiwan; 5 Graduate Institute of Biomedical Informatics, College of Medical Science and Technology, Taipei Medical University, Taipei, Taiwan; 6 Graduate Institute of Clinical Medicine, College of Medicine, National Taiwan University, Taipei, Taiwan

**Keywords:** Out-of-hospital cardiac arrest, Targeted temperature management, Neurological outcome, Neuroprognostication

## Abstract

**Background:**

Neurological outcomes after out-of-hospital cardiac arrest (OHCA) depend on multiple factors, including the patient’s baseline condition and post-arrest management. The SLANT, developed specifically for OHCA survivors treated with targeted temperature management (TTM), requires further validation, particularly in Asian populations.

**Methods:**

This multicenter retrospective cohort study analyzed data from 2016 to 2023, examining demographics, pre-arrest conditions, resuscitation events, and laboratory biomarkers following TTM. The primary outcome was defined as a poor neurological outcome at hospital discharge. Model performance was assessed using the area under the receiver operating characteristic curve. Multivariate logistic regression analysis was used to analyze the included variables.

**Results:**

A total of 448 eligible adult patients were included, of whom 77.9% experienced poor neurological outcomes at discharge. The performance of the current cohort was comparable to that of the original SLANT cohort, achieving an area under the curve of 0.797 (95% confidence interval: 0.746–0.849). All five factors of the SLANT score remained statistically significant in predicting poor neurological outcomes. At a cutoff of ≥ 6.5, the SLANT score demonstrated a specificity of 53.5% and positive predictive value (PPV) of 86.9%. Increasing the cutoff value to 8.5 improved the specificity to 66.7% and the PPV to 89.6%.

**Conclusion:**

The SLANT showed high PPV for predicting poor neurological outcomes at discharge in patients with OHCA undergoing TTM across a multicenter Asian cohort. Combining the score with other neurological assessments is recommended for improved neuroprognostication.

**Graphical Abstract:**

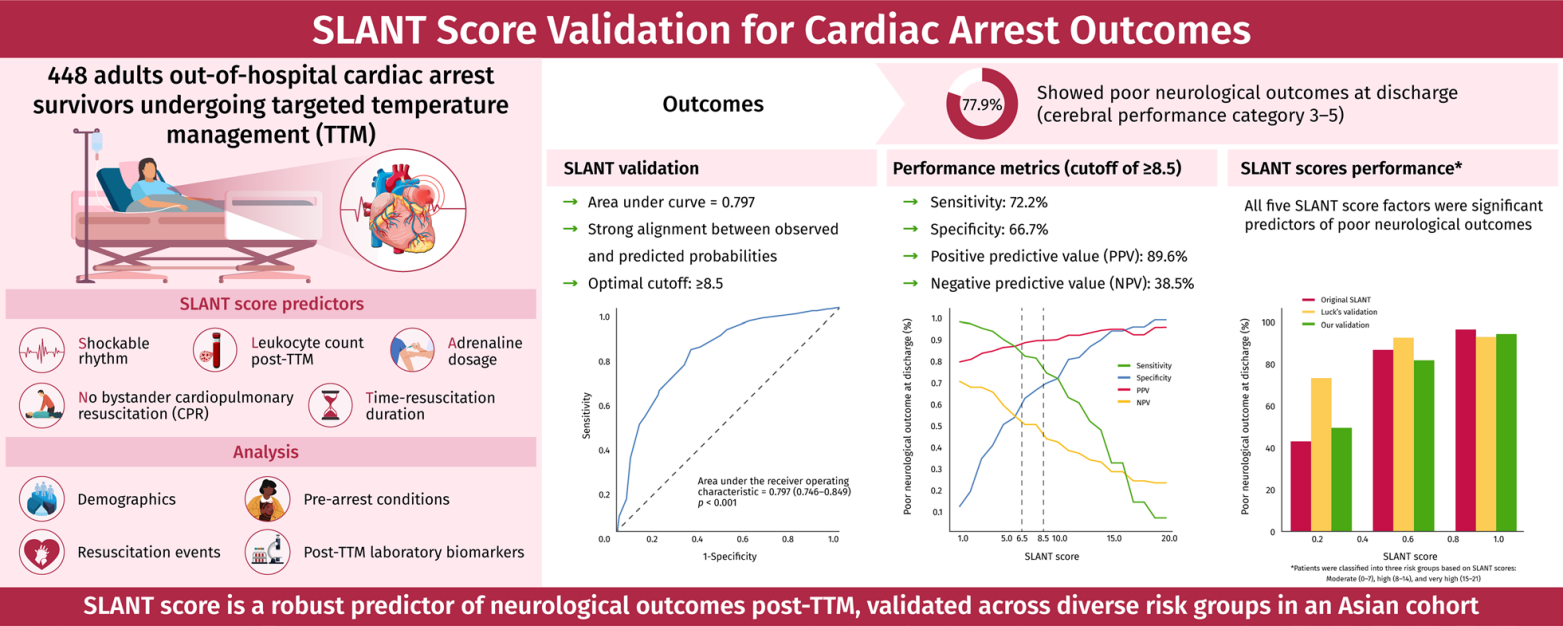

**Supplementary Information:**

The online version contains supplementary material available at 10.1186/s40560-025-00778-y.

## Background

Neurological outcomes following out-of-hospital cardiac arrest (OHCA) are influenced by various factors, including the patient’s baseline condition, resuscitation, and post-arrest management. A recent study in Taiwan reported a significant decline in favorable neurological outcomes, dropping from 13.2% to 9.7% between 2017 and 2021, coinciding with the COVID-19 pandemic [1]. The primary cause of poor neurological outcomes among OHCA survivors is anoxic-ischemic brain injury that occurs during and after resuscitation. Current guidelines recommend targeted temperature management (TTM) within the first 24 h after return of spontaneous circulation (ROSC) to mitigate anoxic brain swelling [2]. Along with optimized post-arrest care, accurate prediction of neurological recovery is essential to guide care and optimize resource allocation. Since 2006, 12 predictive scoring systems have been introduced for neuroprognostication in OHCA survivors [3–14]. Five scoring systems have been developed specifically for comatose OHCA survivors undergoing TTM; however, each has notable limitations [5, 6, 12, 13]. The TTM score only includes patients with a presumed cardiac etiology, which may introduce selection bias [6]. The CAST score was derived from a small cohort of fewer than 100 patients, raising concerns about generalizability [5]. The TIMECARD score omits laboratory variables, making it less comparable to previously published models [13]. In the C-GRApH study, over 87% of poor neurological outcomes were due to death rather than severe disability, raising concerns regarding its predictive accuracy [14].

The SLANT score was recently developed in two large US academic hospitals for adult OHCA survivors treated with TTM between 2018 and 2021 [12]. This model divided patients into three risk groups based on five factors: initial rhythm, post-TTM leukocyte count, adrenaline dosage, witnessed arrest, and resuscitation duration. The score demonstrated good performance, with a C-statistic of 0.852, and was validated in a cohort of 60 OHCA survivors in Taiwan. Luck et al. conducted external validation using a small cohort of 108 patients with in-hospital cardiac arrest (IHCA), in which only 11 patients demonstrated a good neurological outcome [15]. The results showed a moderate predictive power of the SLANT score for neurological status. Along with limitations posed by the small cohort, the characteristics of patients with IHCA differed significantly from those of the OHCA population. The applicability of the SLANT score beyond the original cohort remains uncertain.

Owing to the challenges in collecting data on OHCA survivors treated with TTM, the SLANT score currently lacks comprehensive validation, particularly in Asian populations. Hence, we performed a multicenter study in Taiwan to explore the important factors independent of geographic location, providing updated evidence for neuroprognostication in OHCA survivors undergoing TTM.

## Material and methods

### Study design and setting

This retrospective cohort analysis was based on data collected between January 2016 and December 2023 from the National Taiwan University Hospital (NTUH) and Hsinchu and Yunlin Branch Out-of-Hospital Cardiac Arrest Research Databases in Taiwan. The database enrolled patients from NTUH and its two affiliated hospitals. This study was approved by the Institutional Review Board of NTUH (No. 202401013RINC). The requirement for informed consent was waived because of the retrospective nature of the study.

### Patient selection

Patients meeting the following criteria were included: OHCA survivors with sustained ROSC for more than 20 min, who were admitted to the intensive care unit, completed TTM, were aged 18 years or older, and were unable to follow verbal commands or had a Glasgow Coma Scale (GCS) score ≤ 8 after ROSC.

Patients were excluded if they experienced traumatic arrest, in-hospital mortality during TTM, or were transferred to another hospital during post-arrest care.

A standardized post-arrest care bundle was implemented across the registered hospitals, using cold saline and external cooling devices to lower patients’ core temperatures to 33 °C or 36 °C within 4–6 h of ROSC. The target temperature was maintained for 24 h, followed by rewarming at a rate of 0.25 °C per hour until 36 °C was reached and maintained at 37 °C or lower for an additional 24 h [16]. Absolute contraindications to TTM included catastrophic hemorrhage, severe coagulopathy, refractory ventricular arrhythmias, or premorbid functional status of Glasgow–Pittsburgh Cerebral Performance Category (CPC) 3–5 [17]. Care bundles were guided by ventilator adjustment, metabolic optimization, seizure control, and 12-hourly laboratory biomarkers.

### Data collection and processing

Independent emergency physicians used the Research Electronic Data Capture system [18] to obtain study variables from electronic medical records. To reduce potential biases or errors, individuals involved in data collection were blinded to the study design. Monthly meetings were conducted to address disputed records and ensure consistency in study variables and outcomes.

### Variables

The factors included in the SLANT score were initial rhythm, post-TTM leukocyte count, adrenaline dosage, witnessed arrest, and resuscitation duration.

Demographic variables included age, sex, and body mass index. Resuscitation events included arrest location (home or public), witnessed arrest, bystander cardiopulmonary resuscitation (CPR), initial rhythm (shockable or non-shockable), adrenaline dosage administered during resuscitation in both prehospital and hospital settings, and resuscitation duration. Non-shockable rhythms were classified as asystole, pulseless electrical activity, or bradycardia. Resuscitation duration was defined as the time from the emergency call to sustained ROSC. All resuscitation-related events were collected from medical records using the updated Utstein style template [19, 20]. Pre-arrest comorbidities included coronary artery disease, diabetes mellitus, arrhythmia, heart failure, malignancy, renal insufficiency, and hepatic insufficiency. Post-arrest conditions included reactive pupillary light reflex, GCS motor score, performance of coronary intervention, and whether the patient received extracorporeal membrane oxygenation. Completion of TTM was defined as the full duration of the 24-h hypothermic phase, rewarming phase, and subsequent 24-h normothermic phase. Laboratory data were collected during or after the normothermic phase, including pH, lactate (mmol/L), creatinine (mg/dL), potassium (mEq/L), phosphate (mg/L), hemoglobin (g/dL), leukocyte count (K/μL), and blood urea nitrogen (mg/dL).

### Outcome

The primary outcome was a poor neurological outcome at hospital discharge, defined as a CPC score of 3 to 5. CPC scores were obtained from electronic medical records reviewed by independent physicians.

### Statistical analysis

Continuous variables were assessed for normality using the Kolmogorov–Smirnov test [21] and were expressed as mean (standard deviation) if normally distributed or median (interquartile range) if non-normally distributed. Dichotomous and categorical variables are presented as absolute sample sizes (percentages). Continuous variables were compared using the Mann–Whitney *U* test, whereas dichotomous and categorical variables were examined using the chi-square test.

Missing values in the baseline data were not “completely missing at random.” Unit imputation was applied to address missing values. For continuous variables, including body mass index, total resuscitation duration, and laboratory data after TTM, the median value was imputed. For binary variables, including initial rhythm, reactive pupillary light reflex, and motor score from the GCS, missing values were inputted with “null.”

External validation of the SLANT score was conducted, and discriminatory performance was assessed using the area under the receiver operating characteristic curve (AUC) and corresponding 95% confidence intervals (CIs). Calibration was performed using a calibration plot based on 20 deciles. The Youden index determined the optimal cutoff threshold. Performance metrics, including sensitivity, specificity, positive predictive value (PPV), and negative predictive value (NPV), were calculated with 95% CIs. Positive and negative likelihood ratios were also computed.

Variables were first tested using univariate logistic regression and then adjusted using a multivariate logistic regression model with a backward stepwise approach. Results are reported as adjusted odds ratios (aORs) with 95% CIs. Model fit was assessed using the Hosmer–Lemeshow goodness-of-fit test.

All statistical analyses were performed using SPSS version 26.0 (IBM, Armonk, NY, USA) and R version 4.4.0 (R Foundation for Statistical Computing, Vienna, Austria). The R packages used included ROCR, readxl, survival, epiR, caret, Tidyverse, ggplot2, and CalibrationCurves. A two-sided *p*-value of less than 0.05 was considered statistically significant.

## Results

### Patient enrollment and baseline characteristics

**Fig. 1:**
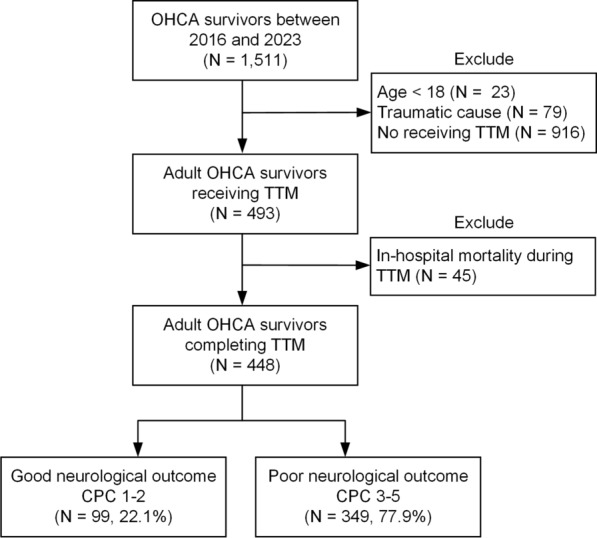
Flowchart of patient selection. *CPC* cerebral performance category, *OHCA* out-of-hospital cardiac arrest, *TTM* targeted temperature management

Figure 1 presents a patient selection flowchart. Initially, 1,511 patients with OHCA who achieved ROSC were screened for eligibility. We excluded 23 patients aged < 18 years, 79 patients with traumatic arrest, and 916 patients who did not receive TTM. Additionally, 45 patients who died during TTM were excluded. Ultimately, 448 adult patients with nontraumatic OHCA treated with TTM were included in the final analysis. Of these, 99 patients (22.1%) had good neurological outcomes at discharge (CPC 1 or 2), whereas 349 patients (77.9%) had poor neurological outcomes (CPC 3–5).

Table 1 presents the baseline characteristics of the study population stratified by neurological outcome. Patients with poor neurological outcomes at discharge were significantly older (66.35 vs. 55.95 years), had more frequent arrests at home locations (47.3% vs. 32.3%), were more likely to present with an initial non-shockable rhythm (70.3% vs. 33.0%), received higher doses of adrenaline (2 mg vs. 0 mg), had longer resuscitation durations (36.90 vs. 26.96 min), and were less likely to receive bystander CPR (45.6% vs. 63.6%). More patients in the poor-outcome group had diabetes or renal insufficiency. Laboratory biomarker levels, including lactate, creatinine, phosphate, leukocyte count, and blood urea nitrogen, were higher in the poor-outcome group, whereas hemoglobin levels were significantly lower. Importantly, patients with poor neurological outcomes had significantly higher SLANT scores (13 vs. 7).

**Table 1:** Baseline demographics and resuscitation variables stratified by neurological outcome

Variables	Total (*n* = 448)	Poor neurological outcome (*n* = 349, 77.9%)	Good neurological outcome (*n* = 99, 22.1%)	*p*
Age	64.05 ± 15.66	66.35 ± 15.26	55.95 ± 14.40	< 0.001
Sex (male)	308 (68.8)	228 (65.3)	80 (80.8)	0.003
BMI (kg/m^2^)	24.28 ± 5.37	24.42 ± 5.70	23.80 ± 4.03	0.353
Arrest location (home)	197 (44.0)	165 (47.3)	32 (32.3)	< 0.001
Witnessed arrest	325 (72.5)	247 (70.8)	78 (78.8)	0.115
Bystander CPR	222 (49.6)	159 (45.6)	63 (63.6)	0.006
Initial nonshockable rhythm	278 (62.1)	245 (70.2)	33 (33.3)	< 0.001
Dosage of adrenalin (mg)	2 [0, 4]	2 [1, 5]	0 [0, 2]	< 0.001
Resuscitation duration (min)	34.70 ± 18.35	36.90 ± 18.72	26.96 ± 14.62	< 0.001
Pre-arrest comorbidities				
Coronary artery disease	106 (23.7)	86 (24.6)	20 (20.2)	0.359
Diabetes mellitus	144 (32.1)	129 (37.0)	15 (15.2)	< 0.001
Arrythmia	47 (10.5)	38 (10.9)	9 (9.1)	0.606
Heart failure	47 (10.5)	35 (10.0)	12 (12.1)	0.549
Malignancy	38 (8.5)	32 (9.2)	6 (6.1)	0.327
Renal insufficiency	57 (12.7)	51 (14.6)	6 (6.1)	0.024
Hepatic insufficiency	7 (1.6)	5 (1.4)	2 (2.0)	0.677
Post-TTM laboratory data				
pH	7.42 ± 0.08	7.42 ± 0.08	7.42 ± 0.05	0.630
Lactate (mmol/L)	2.08 ± 3.26	2.31 ± 3.63	1.25 ± 0.92	0.014
Creatinine (mg/dL)	1.91 ± 1.60	2.09 ± 1.68	1.33 ± 1.15	< 0.001
Potassium (mEq/L)	3.89 ± 0.63	3.92 ± 0.67	3.80 ± 0.46	0.090
Phosphate (mg/L)	3.55 ± 1.75	3.70 ± 1.85	2.87 ± 0.92	0.011
Hemoglobin (g/dL)	10.04 ± 1.99	9.84 ± 1.88	10.72 ± 2.22	< 0.001
Leukocyte (K/uL)	13.50 ± 6.58	14.31 ± 7.09	10.92 ± 3.53	< 0.001
BUN (mg/dL)	33.82 ± 22.94	36.67 ± 23.36	24.24 ± 18.60	< 0.001
Post-arrest neurological findings				
Reactive pupillary light reflex	156 (34.8)	93 (26.6)	63 (63.6)	< 0.001
GCS motor score	1 [1, 4]	1 [1, 2]	4 [1.5, 5]	< 0.001
Post-arrest procedure				
CAG + PCI	88 (19.6)	48 (13.8)	40 (40.4)	0.452
ECMO	43 (9.6)	37 (10.6)	6 (6.1)	0.176
Hospital length of stay (day)	17 [10.25, 29]	17 [10, 31]	17 [13, 23]	0.632
SLANT score	11.66 ± 5.66	13.01 ± 5.01	6.90 ± 5.26	< 0.001

Dichotomous and categorical variables were reported as number (percentages), whereas continuous variables were reported as mean ± standard or median [Q1, Q3]

*BMI* body mass index, *BUN* blood urea nitrogen, *CAG* coronary angiography, *CPR* cardiopulmonary resuscitation, *ECMO* extra-corporeal membrane oxygenation, *GCS* Glasgow coma scale, *mEq* milliequivalent, *mg* milligram, *min* minute, *mmol* millimole, *PCI* percutaneous coronary Intervention, *SLANT*  initial “nonShockable” rhythm, Leukocytosis/Leukopenia” within 24 h after the completion of TTM, total “Adrenalin” dose, lack of “oNlooker” cardiopulmonary resuscitation, and “Time” duration of resuscitation, *TTM*  targeted temperature management

### External validation of the SLANT score

**Fig. 2:**
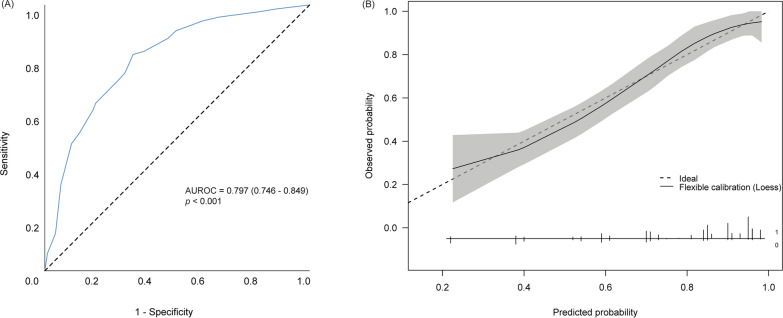
Performance of the SLANT score for prediction probability of poor neurological outcomes **A**) Receiver operating characteristic (R.O.C.) curve for poor neurological outcome. (**B**) Calibration plot of observed versus predicted neurological outcome from validated dataset

The SLANT score included five predictive factors, with initial non-shockable rhythm weighted as the most significant in the original analysis (Supplementary Table 1). The model’s discrimination performance achieved an AUC of 0.797 (95% CI: 0.746–0.849) during external validation (Fig. 2a). The calibration plot demonstrated good alignment between the observed and predicted probabilities of poor neurological outcomes at discharge based on the SLANT score (Fig. 2b).

All five factors of the SLANT score remained statistically significant in predicting poor neurological outcomes. Post-TTM leukocyte count < 4 or > 12 K/µL was the most significant factor (aOR = 4.09, 95% CI 2.43–6.90,* p* < 0.001), followed by initial non-shockable rhythm (aOR = 3.79, 95% CI 2.26–6.36,* p* < 0.001), total adrenaline dose ≧ 5 mg (aOR = 2.56, 95% CI 1.19–5.51, *p* = 0.017), absence of bystander CPR (aOR = 2.29, 95% CI 1.35–3.90, *p* = 0.002), and resuscitation duration ≧ 20 min (aOR = 2.16, 95% CI 1.21–3.86, *p* = 0.010) (Supplementary Table 2).

### Performance of the SLANT score at different cutoff points

Table 2 presents the performance of the SLANT score across three different cohorts: the original SLANT cohort^12^, the validation cohort published by Luck et al. [15], and our cohort. The optimal cutoff point of the score, as determined by the Youden index, was ≥ 6.5, ≥ 8.5, and ≥ 8.5 in each study, respectively. The discrimination performance of our cohort was comparable to that of the SLANT cohort (AUC = 0.797 [95% CI 0.746–0.849] vs. 0.852 [95% CI 0.800–0.903]) and superior to that of Luck’s cohort (AUC = 0.708 [95% CI 0.536–0.879]).

**Table 2:** Performance of SLANT score in predicting poor neurological outcome at discharge

Cohort	Original SLANT	Luck’s validation	Our validation	
AUROC (95% CI)	0.852 (0.800–0.903)	0.708 (0.536–0.879)	0.797 (0.746–0.849)	
Cut-off point	6.5	8.5	6.5	8.5^a^
Sensitivity (95% CI)	84.1	69.1	87.4 (83.5–90.7)	81.4 (76.9–85.3)
Specificity (95% CI)	70.9	72.7	53.5 (43.2–63.6)	66.7 (56.5–75.8)
PPV (95% CI)	89.2^b^	95.7^b^	86.9 (82.9–90.2)	89.6 (85.7–92.7)
NPV (95% CI)	60.9^b^	21.1^b^	54.6 (44.2–64.8)	50.4 (41.5–59.2)
+ LR	2.89	2.53	1.88 (1.52–2.33)	2.44 (1.84–3.24)
− LR	0.22	0.43	0.24 (0.17–0.33)	0.28 (0.22–0.36)

^a^Best cut-off points in the NTUH cohort

^b^Calculated from sensitivity, specificity, and prevalence. PPV = (Sensitivity × Prevalence)/(Sensitivity × Prevalence + (1 − Specificity) × (1 − Prevalence)), NPV = (Specificity × (1 − Prevalence))/(Specificity × (1 − Prevalence) + (1 − Sensitivity) × Prevalence), Prevalence = (Total number with disease)/(Population at risk for the disease)

*CI* confidence interval, *PPV* positive predictive value, *NPV* negative predictive value, + *LR* positive likelihood ratio, − *LR* negative likelihood ratio

In our cohort, when patients were divided into binary groups using a cutoff of ≥ 6.5, the SLANT score achieved a sensitivity, specificity, PPV, NPV, positive likelihood ratio, and negative likelihood ratio of 87.4% (95% CI 83.5%–90.7%), 53.5% (95% CI 43.2%–63.6%), 86.9% (95% CI 82.9%–90.2%), 54.6% (95% CI 44.2%–64.8%), 1.88, and 0.24, respectively (Table 2). When the cutoff was adjusted to ≥ 8.5, the SLANT score showed improved specificity and increased PPV of 66.7% (95% CI 56.5%–75.8%) and 89.6% (95% CI 85.7%–92.7%), respectively.

**Fig. 3:**
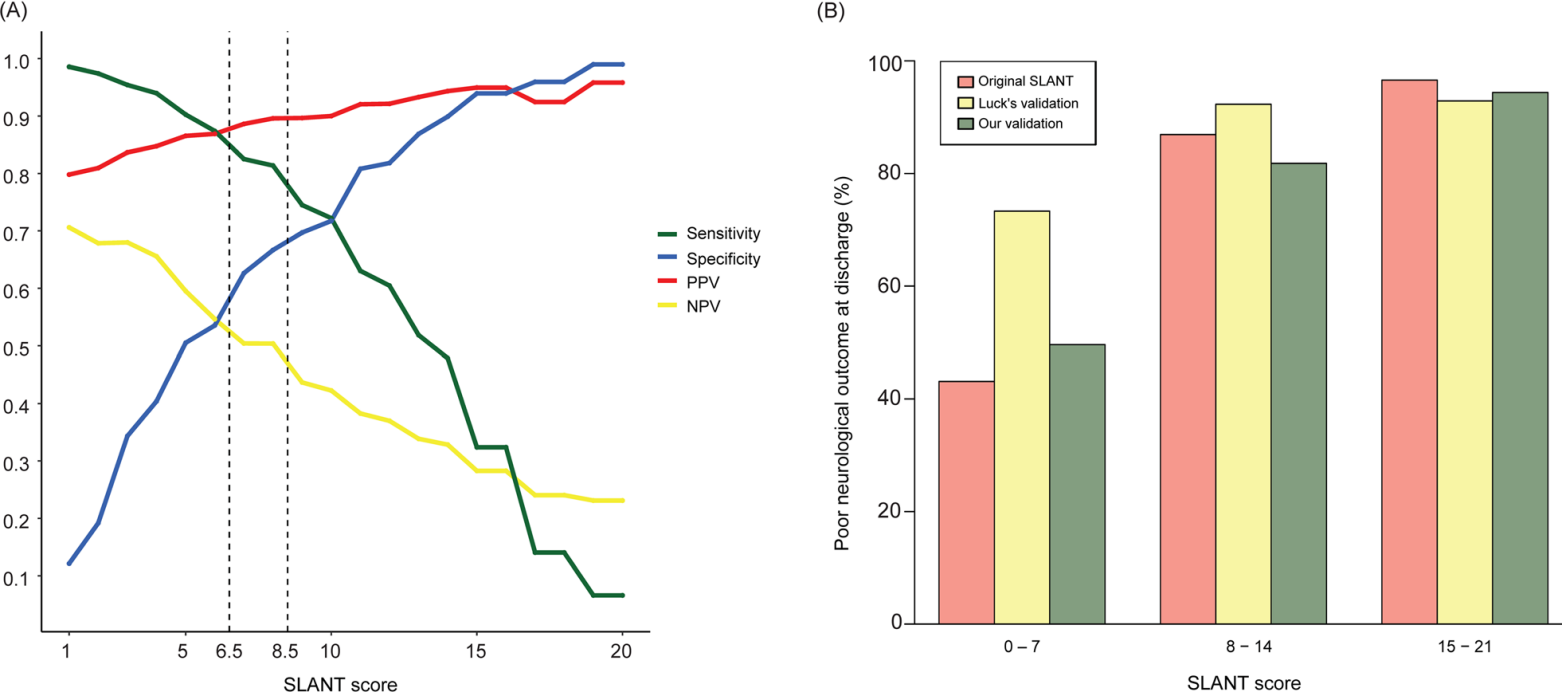
Graphic demonstration of the SLANT score performance. **A** Performance metrics including sensitivity, specificity, positive predictive value, and negative predictive value of SLANT score in our cohort. **B** SLANT scores and risk stratification in the three cohorts (moderate-risk group: 0–7; high-risk group: 8–14; and very high-risk group: 15–21)

Figure 3a presents a graphical representation of the performance metrics. As the SLANT score increases, the sensitivity (green line) sharply decreases, whereas the specificity (blue line) markedly increases. The PPV (red line) shows an upward trend, maintaining consistently high values across the score thresholds, whereas the NPV (yellow line) remains low and exhibits a downward trend as the scores increase. A cutoff value of 8.5 compared to 6.5 offered improved specificity and PPV. Increasing the cutoff to 15 further improved the specificity and PPV to > 90%.

As shown in Fig. 3b, patients were categorized into three risk groups based on their SLANT scores. The probability of poor neurologic outcome at discharge for the moderate-, high-, and very high-risk groups was 43.1%, 86.9%, and 96.6% in the SLANT cohort; 73.3%, 92.3%, and 92.9% in Luck’s cohort; and 49.6%, 81.8%, and 94.4% in our cohort, respectively.

The difference in the percentage of poor neurological outcomes was statistically significant when patients were categorized into either binary risk groups or three-tier risk groups (Supplementary Table 3).

## Discussion

### Brief summary

In this study, the SLANT score demonstrated good calibration and discrimination in predicting neurological outcomes in OHCA patients treated with TTM, comparable to those of the original derivation and validation cohorts, and outperformed the validation cohort reported by Luck et al. [15]. Binary risk stratification using a cutoff of 8.5 points demonstrated a sensitivity, specificity, and PPV of 81.4%, 66.7%, and 89.6%, respectively, for predicting poor neurological outcomes.

### Comparison between the current cohort and the original SLANT cohort

In the current study, all factors from the SLANT score remained significant during logistic regression, with the post-TTM leukocyte count emerging as the strongest predictor. This suggests that the TTM procedure has a substantial impact on outcomes. Compared to the SLANT study population, patients in our cohort were older (64.09 vs. 57.36 years), had a lower incidence of initial shockable rhythm (37.9% vs. 40%), longer resuscitation durations (34.7 vs. 17.0 min), a lower prevalence of coronary artery disease (23.7% vs. 30.2%), and a higher prevalence of diabetes (32.1% vs. 24.6%). Given these differences in baseline characteristics, our cohort was expected to have poorer outcomes; however, the proportion of patients with poor neurological outcomes at discharge was similar between the two cohorts (77.9% vs. 80.0%). Although the predictive performance appears promising in this study, the results may have been overestimated, as the patient characteristics in our cohort tended to be associated with worse outcomes.

To address the potentially overrated performance of the SLANT score, we included all study variables and conducted multivariate logistic regression to explore additional predictive factors (Supplementary Table 4). Resuscitation duration was transformed into a binary factor, with a cutoff value of 27.5 min determined by Youden index analysis. In addition to the SLANT variables, we found that older age (aOR = 1.04, 95% CI 1.02–1.06, *p* < 0.001), poorer motor score on the GCS (aOR = 0.63, 95% CI 0.52–0.77, *p* < 0.001), failure to perform coronary percutaneous intervention (aOR = 0.22, 95% CI 0.11–0.44, *p* < 0.001), and lower levels of hemoglobin after TTM (aOR = 0.81, 95% CI 0.69–0.94, *p* = 0.006) were significantly associated with outcomes. Previous studies have incorporated early motor response as a predictor of neuroprognostication after TTM, all showing moderate discriminatory accuracy (AUC of TTM score = 0.842, AUC of PROLOGUE score = 0.942, AUC of TIMECARD score = 0.885) [6, 11, 13]. The prognostic value of coronary interventions has remained uncertain in studies, as decisions regarding immediate coronary angiography are influenced by clinical judgment. These factors can serve as potential predictors of neuroprognosis and require largescale investigations.

### Comparison between Asian and Eastern population

Geographic and ethnic variations influence the presumed etiology and outcomes of sudden cardiac arrest (SCD). Asians show a lower incidence of SCD compared to African-American, Caucasian, and Hispanic populations, likely due to differences in risk factors such as hypertension, diabetes, smoking, and obesity [22, 23]. However, inherited arrhythmic disorders like Brugada syndrome are more prevalent in Asians [24].

The pre-hospital system also significantly influences OHCA outcomes. Compared to Western world, emergency medical services (EMS) systems in Asia–Pacific countries developed later. The Pan-Asian Resuscitation Outcomes Study (PAROS) [25] reported that, in over 66,000 OHCA cases across seven Asia–Pacific countries, bystander CPR rates ranged from 10.5 to 40.9%, with fewer than 1% receiving bystander defibrillation. Overall survival to discharge was 0.5–8.5%, significantly lower than the 9.6% reported by the North American CARES [26] registry. Moreover, Asian EMS protocols rarely allow pre-hospital death pronouncement, leading to most cases being transported to hospitals for further resuscitation.

The SLANT score heavily emphasizes pre-hospital resuscitation events. However, the lower rates of shockable rhythms, bystander CPR, and defibrillation in Asian OHCA patients directly affect resuscitation duration and outcomes. These regional disparities may lead to an overestimation of the SLANT score’s reliability within the Asian OHCA population, highlighting the need for careful consideration of geographic context in its application.

### Comparison between the current cohort and Luck’s validation cohort

When comparing the external validation process conducted by Luck et al., our cohort presented several key advantages. First, our cohort exclusively enrolled patients with OHCA, which aligned with the inclusion criteria of the original SLANT study, whereas Luck et al. included only patients with IHCA. Since the SLANT score is largely based on variables associated with resuscitation events, its application to an IHCA population may be suboptimal. Second, our study employed a multicenter database, thereby enhancing the robustness and generalizability of the prognostication, as opposed to the single-center design used by Luck et al. Third, the percentage of patients discharged with poor neurological outcomes in our cohort (77.9%) closely mirrored that in the original SLANT study (80.0%) and was lower than that in Luck’s cohort (88.7%). The higher incidence of poor outcomes in the Luck et al. population likely diminished the predictive accuracy for moderate-risk patients and may have compromised the reliability of their validation process.

### Clinical implications

The SLANT score demonstrated good discriminatory performance in a multicenter Asian cohort. It exhibited acceptable specificity, particularly at higher thresholds, for identifying patients prone to poor neurological outcomes. The score maintained a consistently high PPV across all cutoff thresholds. However, a single prediction score is insufficient to provide robust evidence. Combined with clinical, imaging, or laboratory modalities, the SLANT score provides valuable guidance for clinicians and families in deciding on withdrawal of life-sustaining treatment after TTM. It is recommended that clinical physicians utilize this score with a higher cutoff as part of a multimodal approach, incorporating comprehensive post-arrest assessments to identify patients likely to have poor neurological outcomes and ensure the efficient allocation of medical resources.

### Limitations of the original SLANT score

Four of the five factors in the SLANT score are related to resuscitation events, whereas only the leukocyte count is measured after TTM completion. Eight of the 21 points in the SLANT score are attributed to the initial non-shockable rhythm. The score places substantial emphasis on the initial events during cardiac arrest, while giving less consideration to factors determined after ROSC, including early neurological signs or other laboratory biomarkers collected after TTM.

### Limitations in the current study

The current study was conducted across multiple centers and involved over 400 patients, nearly four times the size of the validation cohort of Luck et al., which strengthens the potential for external validation. However, several limitations must be acknowledged.

First, missing data were unavoidable because of the retrospective nature of the study and were not completely missing at random. An imputation process was applied to address the missing values. However, this may not fully mitigate measurement biases associated with missing information. Second, all participating institutions were affiliated with a single leading medical center in Taiwan. This introduced a selection bias, which could affect the generalizability of the results to other settings or populations. An international study involving other Asian countries is recommended. Third, 9.1% of patients were excluded due to in-hospital mortality during TTM, 4.5% higher than in the SLANT study. If these patients had all survived with poor neurological outcomes, the poor outcome rate would increase from 77.9% to 79.9%, aligning with SLANT's 80%. This may enhance the robustness of external validation. However, the small difference still has minimal impact on the overall findings. Finally, the 2020 American Heart Association Guidelines recommend a delayed multimodal approach for neuroprognostication following cardiac arrest [27]. The use of newer modalities, including computed tomography and magnetic resonance imaging, as well as biomarkers, including Neuron-Specific Enolase [28, 29], to quantitatively assess hypoxic-ischemic brain injury may enhance the predictive value of future research.

## Conclusion

In this external validation, the SLANT score demonstrated good performance, showing a high PPV for predicting poor neurological outcomes at discharge in patients with OHCA undergoing TTM in a multicenter Asian population. Combining the SLANT score with other neurological assessments is recommended to enhance neuroprognostication using a multimodal approach.

## Supplementary Information

Supplementary Material 1

## Data Availability

The datasets used and analyzed during the current study are available from the corresponding author on reasonable request.

## Abbreviations

aOR: Adjusted odds ratio
AUC: Area under the curve
CPR: Cardiopulmonary resuscitation
CPC: Cerebral performance category
CIs: Confidence intervals
GCS: Glasgow coma scale
IHCA: In-hospital cardiac arrest
NPV: Negative predictive value
NTUH: National Taiwan University Hospital
OHCA: Out-of-hospital cardiac arrest
PPV: Positive predictive value
ROSC: Return of spontaneous circulation
TTM: Targeted temperature management
