# Supplementary material for: External validation and comparative performance of the SLANT score for neuroprognostication in out-of-hospital cardiac arrest survivors undergoing targeted temperature management: insights from an Asian cohort
Source: J Intensive Care. 2025 Feb 14;13:8. doi: 10.1186/s40560-025-00778-y (PMC11827192; doi:10.1186/s40560-025-00778-y)
Supplement: Supplementary file 1 — Supplementary Material 1 [file 40560_2025_778_MOESM1_ESM.docx]

| **Supplementary Table 1. The weighting of the SLANT score** | |
| --- | --- |
| Predictive Factor | Score assigned |
| Initial nonshockable rhythm | 8 |
| Post target temperature management   Leukocyte count <4 or >12 K/µL | 4 |
| Total adrenalin dose ≧ 5mg | 4 |
| Lack of onlooker cardiopulmonary resuscitation | 2 |
| Resuscitation duration ≧ 20 min | 3 |
| SLANT = initial “nonShockable” rhythm, Leukocytosis/Leukopenia” within 24 hours after the completion of TTM, total “Adrenalin” dose, lack of “oNlooker” cardiopulmonary resuscitation, and “Time” duration of resuscitation. | |

| **Supplementary Table 2. Multivariable logistic regression of the predictive factors included in the SLANT score** | | | | |
| --- | --- | --- | --- | --- |
| Predictive factor | aOR | 95% CI | *p* | Score assigned originally |
| Initial nonshockable rhythm | 3.79 | 2.26 - 6.36 | <0.001 | 8 |
| Post TTM leukocyte count <4 or >12 K/µL | 4.09 | 2.43 - 6.90 | <0.001 | 4 |
| Total adrenalin dose ≧ 5mg | 2.56 | 1.19 - 5.51 | 0.017 | 4 |
| Lack of onlooker cardiopulmonary resuscitation | 2.29 | 1.35 - 3.90 | 0.002 | 2 |
| Resuscitation duration ≧ 20 min | 2.16 | 1.21 - 3.86 | 0.010 | 3 |
| aOR = adjusted odds ratio, CI = Confidence interval, min = minutes, mg = milligram, SLANT = initial “nonShockable” rhythm, Leukocytosis/Leukopenia” within 24 hours after the completion of TTM, total “Adrenalin” dose, lack of “oNlooker” cardiopulmonary resuscitation, and “Time” duration of resuscitation, TTM=targeted temperature management. | | | | |

| **Supplementary Table 3. SLANT scores and risk stratification** | | |
| --- | --- | --- |
| Risk group | Total SLANT score | Poor neurologic status, n (% within risk group) |
| Moderate risk (n=123) | 0 - 7 | 61 (49.6%) |
| High risk (n=148) | 8 - 14 | 121 (81.8%) |
| Very high risk (n=177) | 15 - 21 | 167 (94.4%) |
| *Chi-squared test ( p < 0.001 )* | | |
| Risk group | Total SLANT score | Poor neurologic status, n (% within risk group) |
| Moderate risk (n=131) | 0 - 8 | 65 (49.6%) |
| Higher risk (n=317) | 9 - 21 | 284 (89.6%) |
| *Chi-squared test ( p < 0.001)* | | |
| SLANT = initial “nonShockable” rhythm, Leukocytosis/Leukopenia” within 24 hours after the completion of TTM, total “Adrenalin” dose, lack of “oNlooker” cardiopulmonary resuscitation, and “Time” duration of resuscitation. | | |

| **Supplementary Table 4. Logistic regression analysis of factors associated with poor neurological outcomes at discharge** | | | | | |
| --- | --- | --- | --- | --- | --- |
|  |  | Univariate regression | | Multivariable regression (backward) | |
| Variables | N (%) / Median [IQR] | Crude OR (95% CI) | *p* | Adjusted OR (95% CI) | *p* |
| **Age** | 65.46 [53.27, 75.36] | 1.04 (1.03 - 1.06) | <0.001 | 1.04 (1.02 - 1.06) | <0.001 |
| **Sex (male)** | 308 (68.75) | 0.45 (0.26 - 0.77) | 0.004 |  |  |
| **BMI (kg/m^2^)** | 23.63 [20.83, 27.34] | 1.02 (0.98 - 1.07) | 0.371 |  |  |
| **Arrest location (home)** | 197 (43.97) | 1.88 (1.17 - 3.01) | 0.009 |  |  |
| **Witnessed arrest** | 325 (72.54) | 0.65 (0.38 - 1.11) | 0.117 |  |  |
| **Bystander CPR** | 222 (49.55) | 0.48 (0.30 - 0.76) | 0.002 | 0.34 (0.18 - 0.64) | 0.001 |
| **Initial nonshockable rhythm** | 278 (62.05) | 4.29 (2.48 - 7.41) | <0.001 | 3.63 (1.71 - 7.73) | 0.001 |
| **Dosage of adrenalin (mg)** | 2 [0, 4] | 1.34 (1.19 - 1.51) | <0.001 | 1.25 (1.09 - 1.42) | 0.001 |
| **Resuscitation duration ≧ 27.5 min** | 280 (62.5) | 3.07 (1.94 - 4.87) | <0.001 | 2.04 (1.05 - 3.96) | 0.036 |
| **Pre-arrest comorbidities** |  |  |  |  |  |
| Coronary artery disease | 106 (23.66) | 1.29 (0.75 - 2.23) | 0.360 |  |  |
| Diabetes mellitus | 144 (32.14) | 3.28 (1.81 - 5.93) | <0.001 |  |  |
| Arrythmia | 47 (10.49) | 1.22 (0.57 - 2.62) | 0.607 |  |  |
| Heart failure | 47 (10.49) | 0.81 (0.40 - 1.62) | 0.549 |  |  |
| Malignancy | 38 (8.48) | 1.57 (0.64 - 3.86) | 0.331 |  |  |
| Renal insufficiency | 57 (12.72) | 2.65 (1.10 - 6.38) | 0.029 |  |  |
| Hepatic insufficiency | 7 (1.56) | 0.71 (0.14 - 3.69) | 0.679 |  |  |
| **Post-TTM laboratory data** |  |  |  |  |  |
| pH | 7.43 [7.39, 7.47] | 2.16 (0.10 - 44.73) | 0.619 |  |  |
| Lactate (mmol/L) | 1.43 [1.03, 1.97] | 2.58 (1.58 - 4.22) | <0.001 | 1.52 (0.97 - 2.39) | 0.069 |
| Creatinine (mg/dL) | 1.21 [0.80, 2.50] | 1.53 (1.22 - 1.94) | <0.001 |  |  |
| Potassium (mEq/L) | 3.80 [3.50, 4.28] | 1.39 (0.94 - 2.04) | 0.098 |  |  |
| Phosphate (mg/L) | 3.20 [2.30, 4.30] | 1.40 (1.09 - 1.81) | 0.010 |  |  |
| Hemoglobin (g/dL) | 9.70 [8.60, 11.40] | 0.80 (0.71 - 0.90) | <0.001 | 0.81 (0.69 - 0.94) | 0.006 |
| Leukocyte (K/uL) | 11.94 [9.44, 16.26] | 1.13 (1.07 - 1.20) | <0.001 | 1.10 (1.02 - 1.19) | 0.016 |
| BUN (mg/dL) | 26.80 [17.00, 45.80] | 1.03 (1.02 - 1.05) | <0.001 |  |  |
| **Post-arrest neurological findings** |  |  |  |  |  |
| Reactive pupillary light reflex | 156 (34.82) | 0.21 (0.13 -0.33) | <0.001 |  |  |
| GCS motor score | 1 [1, 4] | 0.54 (0.46 -0.63) | <0.001 | 0.63 (0.52 - 0.77) | <0.001 |
| **Post-arrest procedure** |  |  |  |  |  |
| CAG + PCI | 88 (19.64) | 0.24 (0.14 - 0.39) | <0.001 | 0.22 (0.11 - 0.44) | <0.001 |
| Dichotomous and categorical variables were reported as number (percentages), whereas continuous variables were reported as mean ± standard or median[Q1, Q3]. BMI = body mass index, BUN = blood urea nitrogen, CAG = coronary angiography, COPD = chronic obstructive pulmonary disease, CPR = cardiopulmonary resuscitation, ECMO = extra-corporeal membrane oxygenation, ED = emergency department, GCS = Glasgow coma scale, mEq = milliequivalent, mg = milligram, min = minute, mmol = millimole, PCI = percutaneous coronary intervention, ROSC = return of spontaneous circulation, SLANT = initial “nonShockable” rhythm, Leukocytosis/Leukopenia” within 24 hours after the completion of TTM, total “Adrenalin” dose, lack of “oNlooker” cardiopulmonary resuscitation, and “Time” duration of resuscitation, TTM = targeted temperature management | | | | | |
